# Changes in Gut and Plasma Microbiome following Exercise Challenge in Myalgic Encephalomyelitis/Chronic Fatigue Syndrome (ME/CFS)

**DOI:** 10.1371/journal.pone.0145453

**Published:** 2015-12-18

**Authors:** Sanjay K. Shukla, Dane Cook, Jacob Meyer, Suzanne D. Vernon, Thao Le, Derek Clevidence, Charles E. Robertson, Steven J. Schrodi, Steven Yale, Daniel N. Frank

**Affiliations:** 1 Marshfield Clinic Research Foundation, Marshfield, WI, United States of America; 2 William S. Middleton Memorial Veterans Hospital, Madison, WI, United States of America; 3 University of Wisconsin, Madison, WI, United States of America; 4 Bateman Horne Center of Excellence, Salt Lake City, UT, United States of America; 5 University of Colorado Denver Anschutz Medical Campus, Aurora, CO, United States of America; 6 Marshfield Clinic, Marshfield, WI, United States of America; University of Palermo, ITALY

## Abstract

Myalgic encephalomyelitis/chronic fatigue syndrome (ME/CFS) is a disease characterized by intense and debilitating fatigue not due to physical activity that has persisted for at least 6 months, post-exertional malaise, unrefreshing sleep, and accompanied by a number of secondary symptoms, including sore throat, memory and concentration impairment, headache, and muscle/joint pain. In patients with post-exertional malaise, significant worsening of symptoms occurs following physical exertion and exercise challenge serves as a useful method for identifying biomarkers for exertion intolerance. Evidence suggests that intestinal dysbiosis and systemic responses to gut microorganisms may play a role in the symptomology of ME/CFS. As such, we hypothesized that post-exertion worsening of ME/CFS symptoms could be due to increased bacterial translocation from the intestine into the systemic circulation. To test this hypothesis, we collected symptom reports and blood and stool samples from ten clinically characterized ME/CFS patients and ten matched healthy controls before and 15 minutes, 48 hours, and 72 hours after a maximal exercise challenge. Microbiomes of blood and stool samples were examined. Stool sample microbiomes differed between ME/CFS patients and healthy controls in the abundance of several major bacterial phyla. Following maximal exercise challenge, there was an increase in relative abundance of 6 of the 9 major bacterial phyla/genera in ME/CFS patients from baseline to 72 hours post-exercise compared to only 2 of the 9 phyla/genera in controls (p = 0.005). There was also a significant difference in clearance of specific bacterial phyla from blood following exercise with high levels of bacterial sequences maintained at 72 hours post-exercise in ME/CFS patients versus clearance in the controls. These results provide evidence for a systemic effect of an altered gut microbiome in ME/CFS patients compared to controls. Upon exercise challenge, there were significant changes in the abundance of major bacterial phyla in the gut in ME/CFS patients not observed in healthy controls. In addition, compared to controls clearance of bacteria from the blood was delayed in ME/CFS patients following exercise. These findings suggest a role for an altered gut microbiome and increased bacterial translocation following exercise in ME/CFS patients that may account for the profound post-exertional malaise experienced by ME/CFS patients.

## Introduction

The Centers for Disease Control estimates that between 836,000 and 2.5 million people in the U.S. suffer from myalgic encephalomyelitis/chronic fatigue syndrome (ME/CFS) [1], resulting in substantial disability [2] and significant socio-economic impact [3,4]. ME/CFS is a complex disorder that has been associated with neuro-inflammatory and oxidative processes [5]. One current model of disease suggests that a trigger event (e.g. infection) results in a chronic inflammatory state characterized by increased proinflammatory cytokine production, increased reactive oxygen and nitrogen species, altered intracellular signaling, increased intestinal permeability and systemic activation of innate immune receptors, altered glutaminergic and dopaminergic neurotransmission, mitochondrial dysfunction, and aberrant autoimmune responses [6–11]. These pathogenic processes appear to be self-sustaining and self-amplifying and account for the characteristic symptoms of ME/CFS and other systemic disorders characterized by central and peripheral fatigue [5,9]. Despite recent advances in understanding the biochemical underpinnings of disease, diagnosis continues to rely on clinical presentation [12]. Guidelines established in 1994 by Fukuda et al as part of the International Chronic Fatigue Syndrome Study Group define CFS as clinically evaluated, unexplained, persistent fatigue that is not alleviated by rest that occurs in conjunction with four or more characteristic symptoms, including impaired memory or concentration, sore throat, tender lymph nodes, muscle or joint pain in the absence of redness or swelling, headaches, unrefreshing sleep, and post-exertional malaise (PEM) lasting more than 24 hours, persisting or recurring over 6 or more consecutive months of illness [13]. However, not all patients experience the same symptoms indicating the existence of ME/CFS subgroups. For example, subgroups based on the presence or absence of gastrointestinal symptoms [14] and post-exertional malaise [15] has been described.

In particular, post-exertional malaise has emerged as a distinguishing feature of ME/CFS. It is described as significant worsening of several symptoms following physical and mental exertion. Post-exertional malaise has been associated with abnormal neurovascular regulation [16] and altered immune and metabolic response to aerobic exercise [17–19]. Variability in symptom constellation and severity over time make ME/CFS patients a heterogeneous population and several studies have failed to detect differences between patients and controls at baseline [17,18,20]. As such, exercise challenge in those suffering from PEM may serve as a useful tool for exacerbating symptoms in a controlled setting to attempt to calibrate symptom constellation and severity across the patient group and to uncover potential differences between patients and controls that might not be apparent at rest. Characterization of the gut microbiome in patients with ME/CFS has demonstrated significant alterations compared to healthy controls [21,22]. Additionally, systemic antibody responses to enteric microorganisms have been shown to occur in patients with ME/CFS, suggesting that increased intestinal permeability and bacterial translocation across the intestinal barrier may result in further inflammation and contribute to ME/CFS symptoms [9,23,24]. IgA antibody responses to enteric bacteria in ME/CFS patients have to date been associated with higher serum IL-1, TNFα, and neopterin levels, autoimmune responses to serotonin, and increased symptoms of irritable bowel syndrome [25,26]. We hypothesized that the ecology of the gut microbiota of ME/CFS patients would differ from that of matched healthy controls and that these differences would be associated with increased bacterial translocation from the gut to the circulatory system following exercise challenge with corresponding worsening of symptoms (i.e, pain, fatigue, and mood). The results presented here add further to the previous findings suggesting that ME/CFS patients have an altered gut microbiome and further suggest that increased bacterial translocation following exercise provides a potential explanation for the profound post-exertional malaise experienced by some ME/CFS patients.

## Methods

### Study Subjects

We enrolled 10 ME/CFS patients and 10 healthy controls from the Madison and Marshfield, WI areas from 122 potential subjects responding to study advertisements in local clinics. We were able to reach 104 subjects by phone for screening, 45 of whom did not qualify. By phone, 13 potential patients and 46 potential controls were identified. We enrolled 10 ME/CFS patients and 10 healthy controls from this group for the microbiome study. All patients met the ME/CFS case definition criteria established by Fukuda et al in 1994 [13]. The control group was comprised of healthy people who had no persistent complaints of fatigue. Patients and controls were matched based on age, gender, BMI, and self-reported general activity patterns. Subject age ranged between 20 and 60 years. Each subject gave written consent in an IRB approved consent form to participate in the study. This study was approved by Marshfield Clinic and University of Wisconsin—Madison Institutional Review Boards.

### Inclusion and Exclusion Criteria

Standard medical history was reviewed and a physical exam was conducted for each subject to rule out any major illnesses other than ME/CFS. Routine blood and urine chemistry tests were also used to screen for exclusionary medical conditions or other conditions that may explain the patients’ symptoms. Exclusionary conditions included untreated hypothyroidism, sleep disorders, side effects of medication, relapsing of past medical issues (e.g. Lyme disease, hepatitis B or C), major psychiatric issues, including major depressive disorder with psychotic or melancholic features, alcohol or other substance abuse within two years of the onset of ME/CFS, and severe obesity as defined by a body mass index (BMI) of greater than 40 kg/m^2^. Potential participants were also excluded if they had: 1) current use of immunomodulatory medications, stool softeners, laxatives, anti-diarrheal agents, antibiotics, or probiotics, 2) current use of opioids, 3) a history of cardiovascular disease or uncontrolled hypertension, or 4) current fatigue sufficient to interfere with or preclude exercise testing. Patients were asked to confirm the absence of exclusionary medications on the day of testing and to list other current medication use. Although we did not explicitly exclude for low-dose antidepressant use, gastrointestinal disease, or smoking, which could influence gut flora, none of the participants were currently taking this class of medication, reported any gastrointestinal disorder, or were smokers. Further, none of the participants were diagnosed with depression.

After completion of informed consent, study subjects underwent four non-consecutive days of testing at the Exercise Psychology laboratory at the University of Wisconsin—Madison. Day 1 included a clinical interview and screening blood draw. Day 2 occurred approximately one week later and involved pre-exercise symptom assessment, stool sample collection, maximal exercise test, post-exercise symptom assessment, and blood draws. Expired gases, heart rate, and ratings of perceived exertion and leg muscle pain were collected during exercise and recovery. Participants returned to the laboratory at 48 and 72 hours after exercise to complete self-report symptom questionnaires and provide follow-up stool and blood samples.

### Exercise Test

Participants performed a maximal exercise test on an electronically braked cycle ergometer (Sensormedics, Loma Linda, CA) following the protocol previously described by Cook et al [27]. Seat height was adjusted prior to exercise to the desired fit of the participant. Resistance was software-controlled (Sensormedics, Loma Linda, CA) via an interface cable from a computer to the ergometer. Participants were seated on the stationary bicycle for 1 minute of quiet rest to acquaint themselves to breathing through the mouthpiece and the overall setup. Exercise began with a 3-minute warm-up at 25 Watts (W). Participants were instructed to maintain a pedal rate between 60 and 70 revolutions per minute (RPM) throughout the test. After the warm-up period the work rate was increased by 5 W every 20 seconds (15 W/min) until volitional exhaustion. The exercise test ended when either, 1) the participant could no longer keep up the pedal rate of 60–70 RPMs, 2) the participant stopped pedaling, or 3) some other factor caused us to stop the test (e.g. testing became unsafe for the participant, equipment malfunction). Peak effort was determined based on meeting at least two of the following criteria: 1) respiratory exchange ratio ≥ 1.1, 2) achievement of 85% of age-predicted maximum heart rate, 3) rating of perceived exertion (RPE) ≥ 17, and 4) a change in VO_2_ of <200 ml with an increase in work. Following peak effort, the workload was reduced to 20 W and the participant underwent a three-minute active recovery phase maintaining the pedal rate of 60–70 RPM. After the three-minute recovery period, the participants were provided water *ad libitum*. Participants were guided to a reclined chair for continued recovery. Blood was drawn at 15 minutes post peak effort and immediately followed by questionnaire completion.

### Symptom Assessment

On day 1, participants completed the Centers for Disease Control Symptom Inventory [28] to document the presence, frequency, and severity of the symptoms most commonly reported by ME/CFS patients. On day 1 and all subsequent days, participants completed the 1) McGill Pain Questionnaire—short-form (MPQ) [29], 2) Profile of Mood States (POMS) [30], 3) Fatigue Visual Analog Scale (FVAS) [31], and the 4) Multidimensional Fatigue Inventory (MFI) [32] both before and immediately, 48-hrs, and 72-hrs post exercise.

### Sample Collection

A total of 80 blood samples (four per subject) were collected in PAXgene blood DNA collection tubes (Qiagen) and 60 stool samples (three per subject) were collected separately in single use, disposable, Protocult collection hats followed by self-transference to a 100 ml sterile capped container (abcinc.org). Blood samples were collected at baseline (pre-exercise) and 15 minutes, 48 hours, and 72 hours post-exercise. Stool samples were collected at baseline (pre-exercise) and 48 and 72 hours post-exercise. After collection, stool samples were immediately stored in a freezer at -20°C or on dry ice before being transferred to an additional freezer for long-term storage at -80°C. Frozen samples were sent for laboratory processing on dry ice.

### Molecular Methods

Total genomic DNA was extracted from each clinical sample using the QIAamp DNA Blood Mini Kit, Qiagen, Valencia, CA for blood samples and QIAamp DNA Stool Mini Kit (Qiagen) for stool samples. All DNA samples were quantified using the Qubit DSDNA HS assay kit (Life Technologies, Grand Island, NY) and stored at -80°C until use. All barcoded primers were designed using Roche’s User manual for the 454 sequencing platform and synthesized by Integrated DNA Technologies (Ames, IA). DNA Samples were amplified using a mixture of barcoded 27F forward primers in a 4:1:1:1 ratio (27f-YM:27f-Bif:27f-Bor:27f-Chl) and reverse primer 534R [33]. The 40 μl PCR mixture consisted of 20 pmol forward and reverse primers, 40 μg bovine serum albumin, 20 μl Hot Start mixture, and 2 μl template DNA. The PCR was done in a PE9700 thermocycler (Applied Biosystems, Waltham, MA) with 25 cycles for the stool samples and 40 cycles for the blood samples. Cycling conditions were as follows: initial denaturation at 95°C for 15 min followed by the number of cycles described above (25 or 40) of denaturation at 94°C for 30 seconds, annealing at 60°C for 30 seconds, and elongation at 72°C for 40 seconds, with a final elongation time of 10 minutes. Each amplicon was normalized to 20 ng/μl using the SequalPrep Normalization Kit (Life Technologies, Grand Island, NY). Amplicons were sequenced using the 454 GS FLX+ system using Titanium chemistry (Roche, Branford, CT).

### DNA Sequence Analysis

Polishing and bioinformatic analysis of 16S sequences was performed as previously described [34–37]. In brief, all pyrosequences were screened for nucleotide quality and sequences with bases at 5’ and 3’ ends with mean Q < 20 over a 10 nucleotide window, ambiguous bases with any N residues, or shorter than minimum length (< 300 nucleotides) were discarded. Chimera screening was performed using the *ChimeraSlayer* tool, which requires that sequences be previously aligned with *NAST-iEr* (Hass BJ 2011). Putative chimeras and other sequences that could not be aligned by *NAST-iEr* were removed from subsequent analyses. Genus-level taxonomic calls were produced by the *RDP Classifier* [38], which performs naïve Bayesian taxonomic classification versus a training set. Operational taxonomic units (OTUs) were produced by clustering sequences with identical taxonomic assignments. Relative abundances of OTUs were calculated for each subject by dividing the sequence counts observed for each OTU by the total number of high-quality bacterial sequences generated for the subject. Good’s coverage index was calculated for each sample using the Explicet software package [39].

### Statistical Analysis

Independent samples t-tests were used to determine group differences at baseline and during exercise. A two group (ME/CFS and control) X 3 time (immediately post, 48-hrs post, and 72-hrs post) doubly-multivariate repeated measures MANOVA was used to analyze symptom changes (i.e. post-exertional malaise (PEM)) from baseline. The overall MANOVA compared the groups on a linear combination of symptom variables. This analysis was used to determine the presence of PEM in our ME/CFS participants and is similar to the approach taken previously by our lab [18]. In brief, we chose variables *a priori* to be entered into the model. Variable selection was based on three conditions: 1) the variables were not highly related, 2) they discriminated between patients and controls, and 3) they were responsive to exercise. These choices were made to avoid issues of multicollinearity and artificial inflation of variance explained by the model. With this model we are able to determine whether symptoms are changing over time (i.e. time main effect), whether they differ between ME/CFS and control patients (i.e. group main effect), and whether the changes in symptoms over time differ between the groups (i.e. group X time interaction). In addition to this omnibus test for PEM, we also calculated effect sizes (d) [40] to illustrate the magnitude of each symptom change post-exercise. These analyses were conducted using SPSS for Windows (Version 22.0, Chicago, IL).

We hypothesized that changes in relative abundances of bacterial OTUs in blood samples from baseline to post-exercise challenge would differ between ME/CFS patients and healthy controls. To test this hypothesis for each taxonomic group, we calculated the difference between the pre-exercise OTU relative abundances and the average of the 48 hour and 72 hour post-exercise counts for each individual. The change in sequence read counts was compared between patients at ten taxonomic groups using four different statistical tests: (i) a T-test using 10,000 permutations to obtain P-values to evaluate the significance of the mean differences in count change between patients and controls; (ii) to evaluate the difference in variances (of change in sequence reads) between patients and controls, an F-test was performed, also with 10,000 permutations for the P-values and to compare distribution differences between patients and controls, both (iii) a nonparametric Kolmogorov-Smirnov test was performed (no permutations) and a (iv) Mann-Whitney U test with 10,000 permutations. These analyses were performed using the XLISP-STAT programming language for the permutation routines and Mathematica for the calculation of the Kolmogorov-Smirnov test.

## Results

### Phenotypic Characteristics

The phenotypic characteristics and fatigue measurements of patients and controls at baseline are presented in Table 1. Age, weight, and height were similar for patients and healthy controls and 80% of subjects were female in both groups. The patient group had greater fatigue, less vigor, and scored more poorly on mood symptoms than controls at baseline (p<0.05). Particularly germane to this study, only 3 of the 10 patients and 2 of the 10 controls reported gastrointestinal symptoms and all participants reported mild severity and symptoms present “a little of the time.” All subjects completed a maximal exercise test as described above with results presented in Table 2. Results of maximal exercise testing were similar between the two groups, except that average heart rate was significantly lower in the ME/CFS case group than in the healthy control group.

**Table 1:** Baseline Characteristics of ME/CFS Patients (n = 10) and Healthy Controls (n = 10).

Characteristics	ME/CFS Patients, mean (± SD)	Controls, mean (± SD)
**Age (yrs)**	48.6 (± 10.5)	46.5 (± 13.0)
**Height (in)**	66.6 (± 3.7)	64.4 (± 4.1)
**Weight (lbs)**	150.7 (± 30.3)	144.3 (± 18.3)
**BMI (kg/m** ^**2**^ **)**	23.9 (±4.3)	24.6 (± 3.3)
**Multidimensional Fatigue Inventory (MFI) Scores**		
General	14.6 (± 1.1) ^a^	10.3 (± 1.9)
Physical	15.4 (± 4.0) ^a^	6.8 (± 2.7)
Reduced Activity	14.0 (± 5.3) ^a^	7.6 (± 3.4)
Reduced Motivation	9.4 (± 2.3) ^a^	6.3 (± 2.9)
Mental	14.5 (± 3.4) ^a^	7.6 (± 3.4)
**Profile of Mood States (POMS) Scores**		
Tension	7.5 (± 4.6)	4.4 (± 3.5)
Depression	7.5 (± 10.5)	5.2 (± 9.1)
Anger	4.8 (± 7.2)	3.4 (± 4.7)
Vigor	8.6 (± 3.4) ^a^	18.9 (± 4.9)
Fatigue	14.5 (± 4.8) ^a^	4.8 (± 6.0)
Confusion	9.1 (± 3.6) ^a^	4.7 (± 4.6)
Total Mood Disturbance	134.8 (± 25.6) ^a^	103.6 (± 28.7)
**McGill Pain Questionnaire**	6.8 (± 5.8)	2.5 (± 5.2)
**CDC Symptom Inventory: Abdominal Symptoms**		
Diarrhea	0.6 (± 1.0)	0.3 (± 0.7)
Stomach/Abdominal Pain	1.1 (± 2.1)	0.0
**CDC Symptom Inventory: Neurocognitive Symptoms**		
Memory Problems	4.0 (± 3.0)	0.2 (± 0.7)
Concentration Problems	4.7 (± 2.8)	0.5 (± 1.6)

ME/CFS, myalgic encephalomyelitis/chronic fatigue syndrome; SD, standard deviation.

^a^ Significantly different from control (*p* < 0.05)

**Table 2:** Maximal Exercise Test Results for ME/CFS Patients (n = 10) and Health Controls (n = 10).

Results at Peak Exercise	ME/CFS Patients, mean (± SD)	Controls, mean (± SD)
VO_2peak_ (ml/kg/min)	28.6 (± 9.0)	28.2 (± 9.6)
Respiratory Exchange Ratio	1.19 (± 0.10)	1.20 (± 0.11)
Rating of Perceived Exertion	18.2 (± 2.0)	16.4 (± 2.7)
Leg Muscle Pain	5.6 (± 3.3)	4.2 (± 1.6)
Heart Rate (beats per minute)	159.0 (± 16.5) ^a^	178.8 (± 6.8)
Time to Fatigue (min)	11.7 (± 2.8)	13.1 (± 3.4)

ME/CFS, myalgic encephalomyelitis/chronic fatigue syndrome; SD, standard deviation

^a^ Significantly different from control (*p* < 0.05)

### Post-exertion Malaise

Self-reported symptoms and effect sizes for selected symptom changes pre- to post-exercise for ME/CFS patients and controls are presented in Table 3. For a complete detailed analysis of all symptoms pre- and post-exercise challenge please see Meyer et al [18]. For the doubly-multivariate repeated measures MANOVA, we compared symptom changes for fatigue (Fatigue VAS), pain (MPQ total) and confusion (POMS Confusion subscale) at three points post-exercise (immediate, 48-hrs, and 72-hrs). Results indicated a significant main effect of Time [Wilks’ Λ = 0.408; F(6,68) = 6.416; p = 0.000] and a significant Group x Time interaction [Wilks’ Λ = 0.611; F(6,68) = 3.163; p = 0.009], showing that symptoms were changing from pre- to post-exercise and that these changes were different between ME/CFS and control patients. Effect size calculations showed that ME/CFS patients had large changes in their symptoms of pain, fatigue, and confusion at various times post-exercise compared to controls.

**Table 3:** Symptom Responses to Maximal Exercise for ME/CFS Patients (n = 10) and Healthy Controls (n = 10).

		Group Means (±SD)				Effect sizes (*d*) for change scores		
Measure	Group	Pre-Ex	Post-Ex	48-hr	72-hr	Post-Ex	48-hr	72-hr
Fatigue VAS	ME/CFS	50.4 (23.3)	67.1 (15.0)	59.5 (23.7)	57.3 (20.0)	0.25	0.91	0.96
	Control	12.3 (12.7)	25.0 (14.5)	7.9 (8.5)	4.1 (6.3)	-	-	-
MPQ Total	ME/CFS	6.9 (5.8)	10.2 (6.9)	7.8 (6.1)	5.4 (4.5)	1.23	0.44	-0.31
	Control	0.8 (1.1)	1.4 (1.1)	0.6 (0.8)	0.2 (0.4)	-	-	-
POMS Confusion	ME/CFS	9.1 (3.6)	12.6 (3.9)	11.3 (4.0)	11.1 (4.9)	1.26	0.85	1.16
	Control	4.1 (3.3)	3.3 (3.1)	3.7 (2.7)	2.4 (2.0)	-	-	-

ME/CFS, myalgic encephalomyelitis/chronic fatigue syndrome; SD, standard deviation; VAS, visual analog scale; MPQ, McGill Pain Questionnaire; POMS, Profile of Mood States; Ex, exercise.

### Microbiome Characteristics

A total of 406,880 high quality sequencing reads were generated with a median of 2,160 reads per specimen. The sequencing coverage at the genus level as estimated by Good’s coverage mean index was 95% for sequence libraries from all individual blood and stool samples, indicating that each sequence dataset adequately captured the underlying biodiversity in the sample from which it was generated. The blood samples, as expected, yielded a lower number of bacterial sequences (n = 111,492; 1,394 per sample) than the stool samples (n = 295,388; 4,923 reads per sample).

Average relative abundances of the bacterial taxa observed in all blood and stool samples collected from patients and controls are shown in Table 4 with pooling for all time points. In blood samples, there was a lower relative abundance of *Bacteroidetes* and higher relative abundance of *Firmicutes* observed in ME/CFS patients than in healthy controls. In contrast, in the stool samples there was a higher relative abundance of *Bacteroidetes* and lower abundance of *Firmicutes* observed in ME/CFS patients compared to healthy controls. The relative abundance of *Actinobacteria* in the gut was significantly lower in ME/CFS patients than healthy controls by a Kolmogorov-Smirnov test (p = 0.007). However, differences were not found to be statistically significant for other bacterial taxa in the blood or stool samples using a non-parametric test to account for both prevalence and abundance [41].

**Table 4:** Relative Abundance of Bacterial Phyla in Blood and Stool Samples from ME/CFS Patients and Health Controls.

	Blood Samples^a^		Stool Samples^b^	
Phyla	ME/CFS Patients	Controls	ME/CFS Patients	Controls
*Actinobacteria*	10.52%	10.80%	0.58%^c^	1.06%
*Bacteroidetes*	17.84%	22.38%	27.71%	22.43%
*Firmicutes*	9.52%	7.89%	58.40%	65.29%
Other	7.97%	8.55%	9.71%	9.11%
*Proteobacteria*	54.14%	50.38%	3.59%	2.12%

ME/CFS, myalgic encephalomyelitis/chronic fatigue syndrome.

^a^ Mean relative abundance for 10 case and 10 control samples collected at baseline and 15 min, 48 hours, and 72 hours post-exercise challenge.

^b^ Mean relative abundance for 10 case and 10 control samples collected at baseline and 48 and 72 hours post-exercise challenge.

^c^ Significantly different from control (*p* < 0.05)

### Microbiome Response to Maximal Exercise

**Fig 1:**
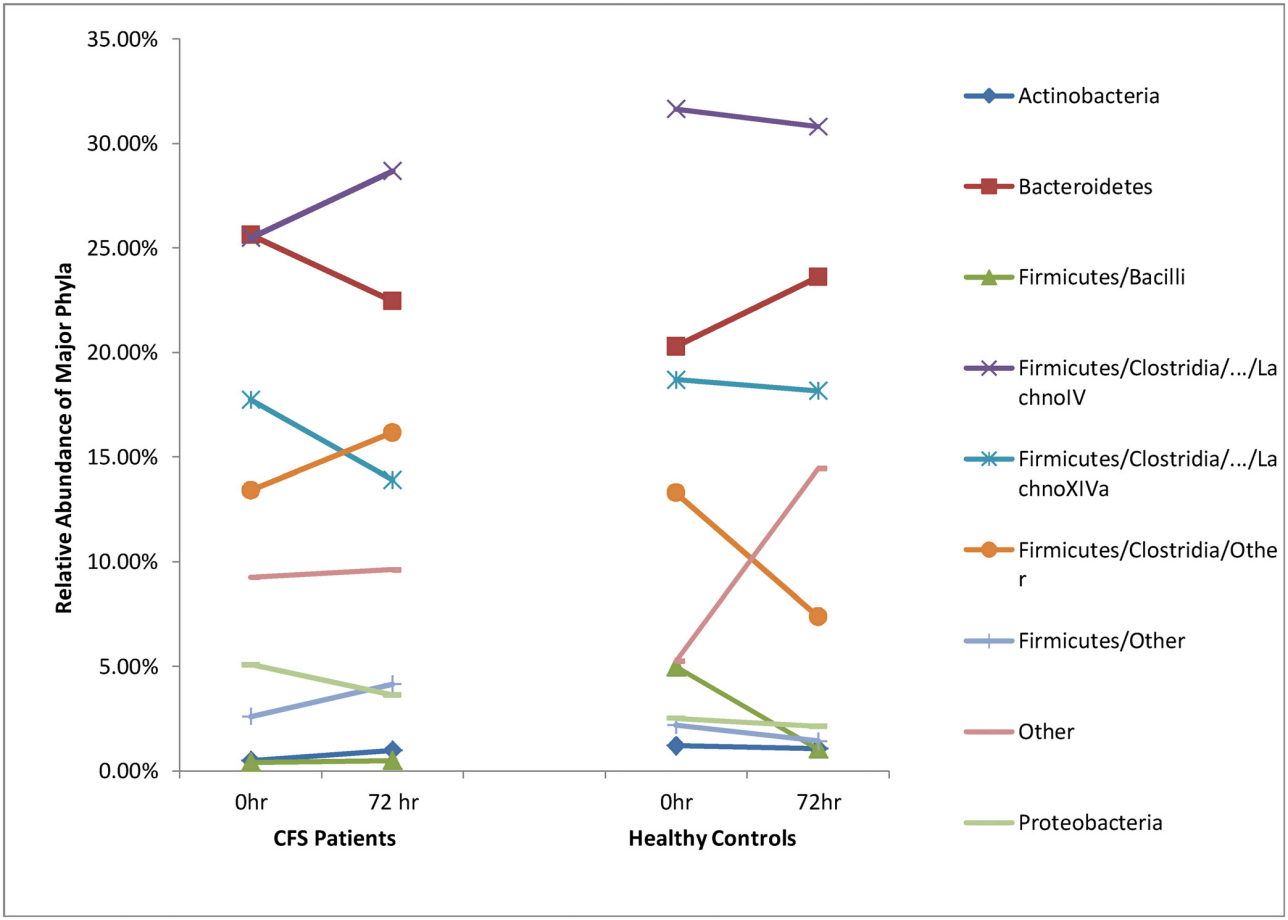
Relative abundance of the major bacterial taxa in stool samples collected at baseline (0 hr) and 72 hr after exercise challenge in ME/CFS patients and healthy controls.

Changes in average relative abundances of bacterial taxa in stool samples were observed following maximal exercise testing and these changes were different in ME/CFS patients and healthy controls. The average relative abundance of 7 out of 9 major taxa increased in the stool in patients from baseline to 72 hours post-exercise compared to an increase in only 2 of the 9 major phyla/genera in healthy controls (p = 0.005) (Fig 1). In contrast to the ME/CFS patients, the relative abundance of most major phyla decreased at 72 h in the stool samples from healthy controls suggesting that the bacterial load in ME/CFS patients is preferentially enhanced during post-exertional malaise.

**Fig 2:**
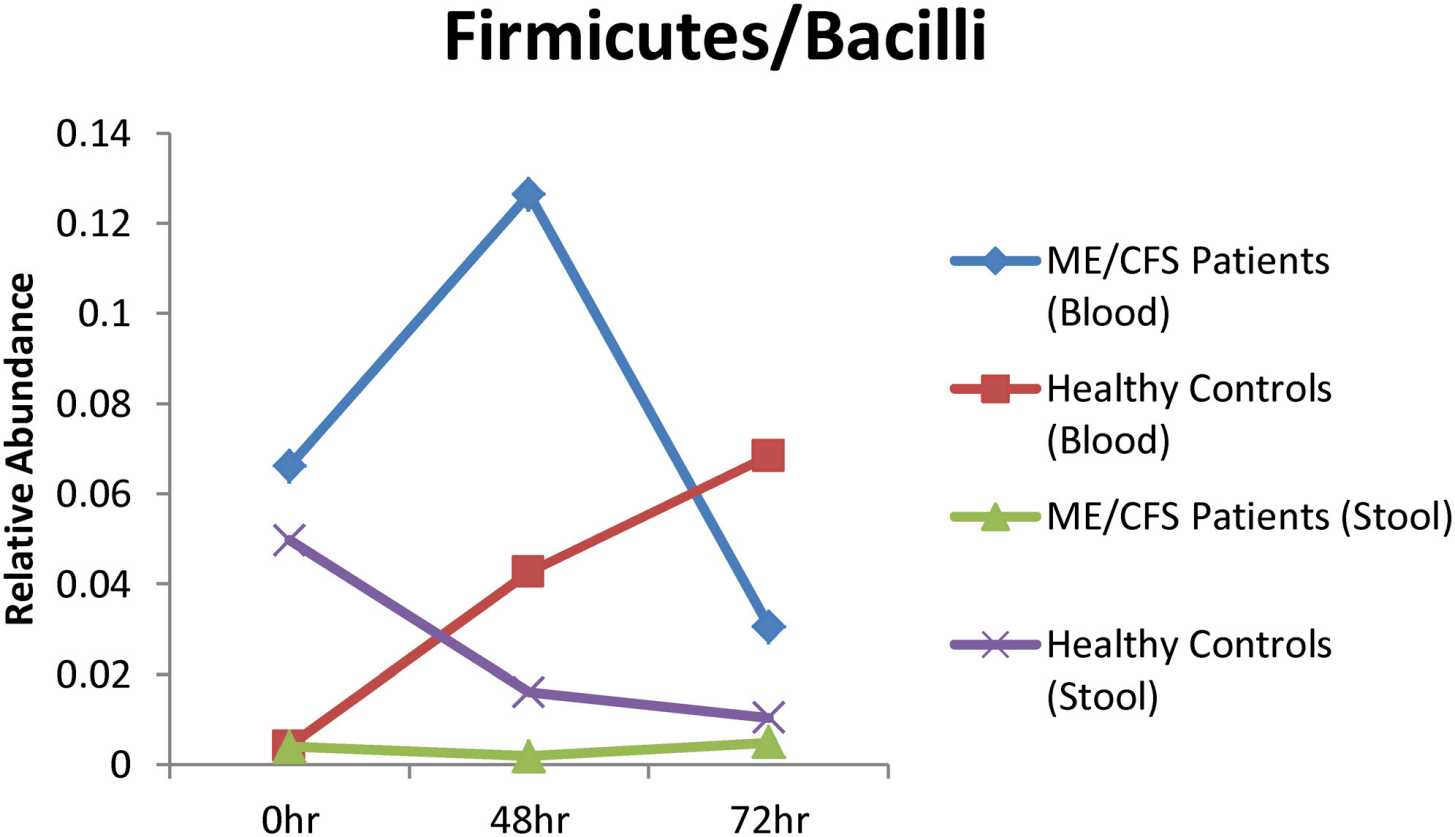
Changes in the relative abundance of *Firmicutes/Bacilli* in blood and stool samples before (0 hr) and after maximal exercise.

**Fig 3:**
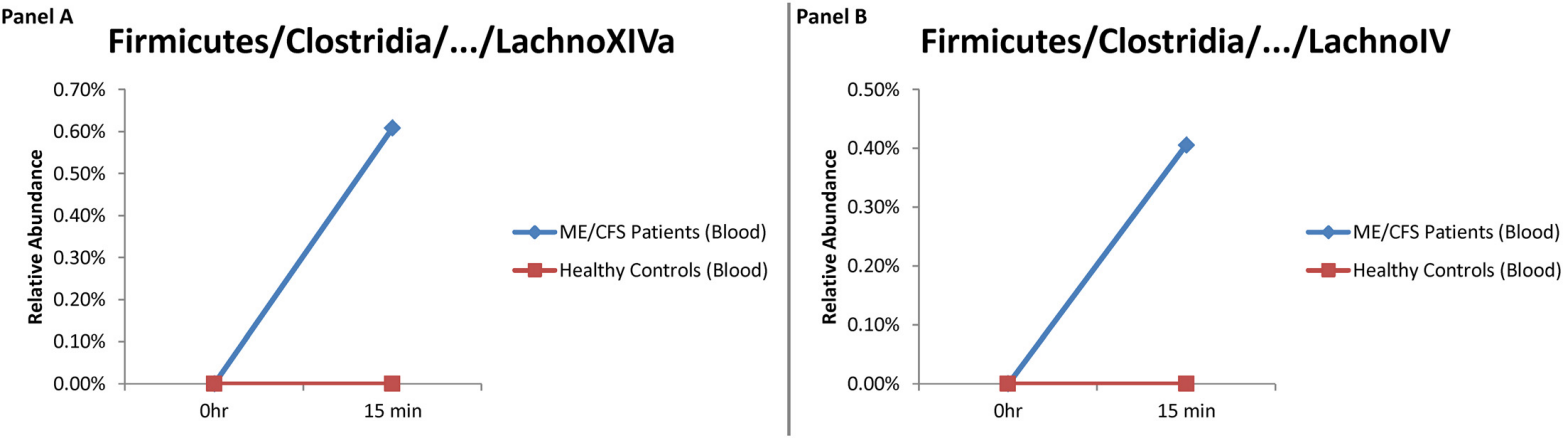
Relative abundance of (A) *Firmicutes/Clostridia/…/LachnoXIVa* and (B) *Firmicutes/Clostridia/…/LachnoIV* in blood samples collected at baseline and 15 minutes after exercise challenge in ME/CFS patients and healthy controls.

Given the high relative abundance of *Firmicutes* in stool samples, we further assessed the potential for translocation of organisms from this phylum into the bloodstream after exercise challenge. Differential changes in the relative abundance of *Firmicutes/Bacilli* organisms were observed in blood and stool samples over time (Fig 2). Of note is the significant increase in the relative abundance of *Bacilli* in blood samples collected from ME/CFS patients at the 48 hour time point. We also observed rapid changes in the relative abundances of the *Clostridium XIVa* and *IV* clusters, belonging to the phylum *Firmicutes*, in blood samples collected 15 min after maximal exercise from ME/CFS patients, but not healthy controls (Fig 3). We speculate that these bacteria may have translocated into the blood stream from the gut after the maximal exercise challenge. This phenomenon appears to be specific to particular taxa and more prominent in patients than controls.

## Discussion

Neuroinflammation and oxidative dysregulation have been shown to occur in patients with ME/CFS [5]. Although, differential intestinal microbiome characteristics have been described for ME/CFS patients and healthy controls [21] and systemic antibody responses to enteric bacteria have been associated with increased inflammation, worsening fatigue, and gastrointestinal symptoms [25,26], the potential for transient changes in bacterial colonization in the gut and/or bloodstream to modulate symptomology has not been evaluated in ME/CFS patients. The evidence of altered intestinal microbiota and bacterial translocation into the bloodstream following exercise challenge in patients with ME/CFS is consistent with previous findings and provides novel evidence of systemic bacterial signal and exercise induced bacterial translocation—one potential explanation for the worsening of symptoms seen in patients when they attempt to become more physically active.

Over the last several years our understanding of how alterations in the human microbiome influence health and disease has increased considerably [42,43]. Murine models suggest that the gut microbiota could contribute to leanness, obesity, stress, and emotional behavior through endocrine and neuroendocrine pathways [44] and considerable evidence suggests similar effects in humans via effects on host immunity and metabolism [45,46]. The differences in the relative abundance of bacteria making up the intestinal microbiome noted in the present study are consistent with those previously reported in the literature [21]. Although no “typical” enterotype has been defined to date, reports of dysbiosis in patients with ME/CFS in Norway and Belgium are consistent with the changes in relative abundance of *Firmicutes* and *Bacteroidetes* observed here [21].

Perhaps more important than confirmation of intestinal dysbiosis in the context of ME/CFS is the evidence of temporal changes in microbiome composition following maximal exercise challenge and the novel finding of bacterial signal in the bloodstream of both ME/CFS patients and healthy controls occurring concomitantly with symptom exacerbation. This finding is consistent with the systemic response to enteric microorganisms reported by Maes et al in a number of reports and demonstration of correlation between this systemic response and pathological ME/CFS processes and symptoms, including higher serum IL-1, TNFα, and neopterin levels, autoimmune responses to serotonin, and increased symptoms of irritable bowel syndrome [9,23–26]. In the present study, changes in microbiome and bacterial measures were accompanied by large changes in fatigue, pain, and confusion in ME/CFS patients. Such exercise-induced changes in the microbiome are consistent with the changes described above and additional recent reports of altered immune response to exercise in patients with ME/CFS, while also providing a plausible mechanistic explanation for such alterations [19].

Although intestinal microbiome composition is known to change over time with development and aging as well as in response to dietary changes, temporal examination of the human intestinal microbiome generally spans weeks, months, or even years to assess evidence of an intervention’s effect. The notion that exercise might influence gut microbiota composition has been described in both animal and human models [47–51]. The evidence presented here suggests that not only does physical activity influence gut microbiota composition, but that the temporal effects of such physical activity may manifest differently in healthy and diseased individuals. These changes are one potential explanation for why acute exercise may make some individuals with ME/CFS sicker.

Blood is generally considered sterile, although evidence of transient, asymptomatic bacteremia has been reported following dental extraction [52] and intestinal insult [53]. In the context of ME/CFS, systemic responses to gut microorganisms suggest that bacterial translocation across the intestinal barrier may also occur as part of this disease [9,23–26]. The notion that exercise may also result in translocation of bacteria across the intestinal barrier is particularly interesting, especially in the case of ME/CFS where post-exertional malaise can be a key characteristic of the disease. Following maximal exercise testing, we detected bacterial signal in blood samples from both ME/CFS patients and healthy controls. Consistent with differences in the intestinal microbiomes between the two groups, we noted an increased relative abundance of *Firmicutes*, particularly those from *Clostridium* clusters *XIVa* and *IV*, in blood samples from ME/CFS patients at 15 minutes post-exercise challenge. *In vitro* functional studies will be able to address this observation better. However, we speculate that some members of Firmicutes and Bacilli because of their stronger cell walls and inherent ability to survive in harsher environmental conditions may have contributed to it surviving longer in bloodstream. Further investigation of the potential for transient translocation of intestinal microorganisms into the bloodstream following exercise and how the dysbiosis characteristic of certain disease states, such as ME/CFS, might influence this translocation may provide considerable insight into how the microbiome influences disease symptoms.

Evidence for altered intestinal permeability in patients with ME/CFS has been mounting [9] and preliminary studies suggest that treatments designed to modulate the gut microbiota or enhance intestinal barrier function may be able to improve ME/CFS symptoms [24,54–56]. Our ability to detect changes in intestinal microbiome composition over time and to observe what appears to be transient bacterial translocation from the gut into the bloodstream following exercise challenge may provide a protocol for testing future treatments designed to alter these outcomes and to determine whether this is the mechanism of action for such treatments. Treatment paradigms that have been tested with some success for other chronic, inflammatory, non-communicable diseases thought to be related to intestinal dysbiosis include probiotics, prebiotics, dietary fiber, and fecal microbiotia transplantation [46,57]. Similar trials in ME/CFS patients may benefit from temporal monitoring for bacterial signal in both the gastrointestinal tract and bloodstream.

Proper diagnosis of ME/CFS is a very involved and thorough process. One of the strengths of this study was that clinicians with expertise in diagnosing ME/CFS were engaged in identifying both patients and controls from a cohort of more than 100 possible patients. Identified patients were included only after examining results of screening blood tests to rule out any major comorbid conditions that could explain the ME/CFS symptoms and controls were carefully selected for matching based on age, gender, BMI, and self-reported general activity patterns. However, given that this was a small pilot study with only 10 patients and 10 controls it was not possible to control for all potential confounders, including the broad age range of participants, historical use of medications and supplements (e.g. pain killers, antioxidants), and additional medical diagnoses (e.g. depression, gastrointestinal symptoms, allergies). While the careful selection process allowed for high quality case and control populations, the study sample size was small and as such, many observations failed to rise to the level of statistical significance. Additionally, the small sample size precluded us from directly examining the associations between symptoms and changes to the gut and plasma microbiome. Given these limitations, findings must be interpreted with caution. An increase in sample size would help us to better assess clinically relevant observations. However, even this relatively small study points to important temporal differences in intestinal microbiome composition and transient bacteremia that will inform future larger studies designed to understand how these differences relate to ME/CFS etiology and/or symptomology. Another study limitation was in the depth of microbiome sequencing. Our approach did satisfy the Good’s coverage index of >0.95, but additional deep sequencing of the samples already collected would likely increase the statistical power to detect significant changes in the rarer bacterial taxa.

We are still a long way from fully understanding how the intestinal microbiota impacts etiology and symptomology in ME/CFS, but the evidence presented here and elsewhere suggests that changes in gut microbiome are associated with this disease. Here, we present additional evidence to support the idea that temporal changes in microbial composition in the gut and translocation of gut microbes into the bloodstream may influence the symptoms of ME/CFS. Future studies of ME/CFS etiology and treatment approaches should incorporate temporal microbial analyses to further elucidate this interesting finding.

## References

[1] Centers for Disease Control and Prevention. Chronic Fatigue Syndrome (CFS): Who’s at Risk? Available: http://www.cdc.gov/cfs/causes/risk-groups.html.

[2] Solomon L, Nisenbaum R, Reyes M, Papanicolaou DA, Reeves WC. Functional status of persons with chronic fatigue syndrome in the Wichita, Kansas, population. Health Qual Life Outcomes. 2003;1: 48 14577835 10.1186/1477-7525-1-48 PMC239865

[3] Jason LA, Benton MC, Valentine L, Johnson A, Torres-Harding S. The economic impact of ME/CFS: individual and societal costs. Dyn Med. 2008;7: 6 10.1186/1476-5918-7-6 18397528 PMC2324078

[4] Reynolds KJ, Vernon SD, Bouchery E, Reeves WC. The economic impact of chronic fatigue syndrome. Cost Eff Resour Alloc. 2004;2: 4 15210053 10.1186/1478-7547-2-4 PMC449736

[5] Morris G, Berk M, Galecki P, Walder K, Maes M. The neuro-immune pathophysiology of central and peripheral fatigue in systemic immune-inflammatory and neuro-immune diseases. Mol Neurobiol. 2015 1 20. [Epub ahead of print] 10.1007/s12035-015-9090-9 25598355

[6] Maes M. Inflammatory and oxidative and nitrosative stress pathways underpinning chronic fatigue, somatization and psychosomatic symptoms. Curr Opin Psychiatry. 2009;22: 75–83. 19127706 10.1097/yco.0b013e32831a4728

[7] Maes M, Twisk FN. Chronic fatigue syndrome: Harvey and Wessley’s (bio)psychosocial model versus a bio(psychosocial) model based on inflammatory and oxidative and nitrosative stress pathways. BMC Med. 2010;8: 35 10.1186/1741-7015-8-35 20550693 PMC2901228

[8] Morris G, Maes M. A neuro-immune model of myalgic encephalomyelitis/chronic fatigue syndrome. Metab Brain Dis. 2013;28: 523–540. 10.1007/s11011-012-9324-8 22718491

[9] Morris G, Maes M. Oxidative and nitrosative stress and immune-inflammatory pathways in patients with myalgic encephalomyelitis (ME)/chronic fatigue syndrome (CFS). Curr Neuropharmacol 2014;12: 168–185. 10.2174/1570159X11666131120224653 24669210 PMC3964747

[10] Morris G, Berk M, Galecki P, Maes M. The emerging role of autoimmunity in myalgic encephalomyelitis/chronic fatigue syndrome (ME/cfs). Mol Neurobiol. 2014;49: 741–756. 10.1007/s12035-013-8553-0 24068616

[11] Morris G, Maes M. Mitochondrial dysfunctions in myalgic encephalomyelitis/chronic fatigue syndrome explained by activated immuno-inflammatory, oxidative and nitrosative stress pathways. Metab Brain Dis. 2014;29: 19–36. 10.1007/s11011-013-9435-x 24557875

[12] Morris G, Maes M. Case definitions and diagnostic criteria for myalgic encephalomyelitis and chronic fatigue syndrome: from clinical-consensus to evidence-based case definitions. Neuro Endocrinol Lett. 2013;34: 185–199. 23685416

[13] Fukuda K, Straus SE, Hickie I, Sharpe MC, Dobbins JG, Komaroff A, et al The chronic fatigue syndrome: a comprehensive approach to its definition and study. Ann Intern Med. 1994;121: 953–959. 7978722 10.7326/0003-4819-121-12-199412150-00009

[14] Maes M, Leunis JC, Geffard M, Berk M. Evidence for the existence of myalgic encephalomyelitis/chronic fatigue syndrome (ME/CFS) with and without abdominal discomfort (irritable bowel) syndrome. Neuro Endocrinol Lett. 2014;35: 445–453. 25433843

[15] Maes M, Twisk FN, Johnson C. Myalgic encephalomyelitis (ME), chronic fatigue syndrome (CFS), and chronic fatigue (CF) are distinguished accurately: results of supervised learning techniques applied on clinical and inflammatory data. Psychiatry Res. 2012;200: 754–760. 10.1016/j.psychres.2012.03.031 22521895

[16] Cook DB, Nagelkirk PR, Peckerman A, Poluri A, Lamanca JJ, Natelson BH. Perceived exertion in fatiguing illness: civilians with chronic fatigue syndrome. Med Sci Sports Exerc. 2003;35: 563–568. 12673137 10.1249/01.MSS.0000058360.61448.6C

[17] Light AR, White AT, Hughen RW, Light KC. Moderate exercise increases expression for sensory, adrenergic, and immune genes in chronic fatigue syndrome patients but not in normal subjects. J Pain. 2009;10: 1099–1112. 10.1016/j.jpain.2009.06.003 19647494 PMC2757484

[18] Meyer J, Light A, Shukla SK, Clevidence D, Yale S, Stegner AJ, et al Post-exertion malaise in chronic fatigue syndrome: symptoms and gene expression. Fatigue: Biomedicine, Health, & Behavior. 2013;1: 190–209.

[19] Nijs J, Nees A, Paul L, De Kooning M, Ickmans K, Meeus M, et al Altered immune response to exercise in patients with chronic fatigue syndrome/myalgic encephalomyelitis: a systematic literature review. Exerc Immunol Rev. 2014;20: 94–116. 24974723

[20] Natelson BH, Haghighi MH, Ponzio NM. Evidence for the presence of immune dysfunction in chronic fatigue syndrome. Clin Diagn Lab Immunol. 2002;9: 747–752. 12093668 10.1128/CDLI.9.4.747-752.2002 PMC120010

[21] Frémont M, Coomans D, Massart S, De Meirleir K. High-throughput 16S rRNA gene sequencing reveals alterations of intestinal microbiota in myalgic encephalomyelitis/chronic fatigue syndrome patients. Anaerobe. 2013;22: 50–56. 10.1016/j.anaerobe.2013.06.002 23791918

[22] Lakhan SE, Kirchgessner A. Gut inflammation in chronic fatigue syndrome. Nutr Metab (Lond). 2010;7: 79. 20939923 10.1186/1743-7075-7-79 PMC2964729

[23] Maes M, Leunis JC. Normalization of leaky gut in chronic fatigue syndrome (CFS) is accompanied by a clinical improvement: effects of age, duration of illness and the translocation of LPS from gram-negative bacteria. Neuro Endocrinol Lett. 2008;29: 902–910. 19112401

[24] Maes M, Mihaylova I, Leunis JC. Increased serum IgA and IgM against LPS of enterobacteria in chronic fatigue syndrome (CFS): indication for the involvement of gram-negative enterobacteria in the etiology of CFS and for the presence of an increased gut-intestinal permeability. J Affect Disord. 2007;99: 237–240. 17007934 10.1016/j.jad.2006.08.021

[25] Maes M, Twisk FN, Kubera M, Ringel K, Leunis JC, Geffard M. Increased IgA responses to the LPS of commensal bacteria is associated with inflammation and activation of cell-mediated immunity in chronic fatigue syndrome. J Affect Disord. 2012;136: 909–917. 10.1016/j.jad.2011.09.010 21967891

[26] Maes M, Ringel K, Kubera M, Anderson G, Morris G, Galecki P, Geffard M. In myalgic encephalomyelitis/chronic fatigue syndrome, increased autoimmune activity against 5-HT is associated with immune-inflammatory pathways and bacterial translocation. J Affect Disord. 2013;150: 223–230. 10.1016/j.jad.2013.03.029 23664637

[27] Cook DB, Stegner AJ, Nagelkirk PR, Meyer JD, Togo F, Natelson BH. Responses to exercise differ for chronic fatigue syndrome patients with fibromyalgia. Med Sci Sports Exerc. 2012;44: 1186–1193. 10.1249/MSS.0b013e3182417b9a 22157881 PMC3319493

[28] Wagner D, Nisenbaum R, Heim C, Jones JF, Unger ER, Reeves WC. Psychometric properties of the CDC Symptom Inventory for assessment of chronic fatigue syndrome. Popul Health Metr. 2005;3: 8 16042777 10.1186/1478-7954-3-8 PMC1183246

[29] Melzack R. The short-form McGill pain questionnaire. Pain. 1987;30: 191–197. 3670870 10.1016/0304-3959(87)91074-8

[30] McNair DM, Lorr M, Droppleman LF. Profile of mood states: manual. San Diego, California: Educational and Industrial Testing Services; 1992.

[31] O’Connor PJ. Mental energy: assessing the mood dimension. Nutr Rev. 2006;64: S7–S9. 16910215 10.1111/j.1753-4887.2006.tb00256.x

[32] Smets EM, Garssen B, Bonke B, Haes JC. The multidimensional fatigue inventory (MFI) psychometric qualities of an instrument to assess fatigue. J Psychosom Res. 1995;39: 315–325. 7636775 10.1016/0022-3999(94)00125-o

[33] Frank JA, Reich CI, Sharma S, Weisbaum JS, Wilson BA, Olsen GJ. Critical evaluation of two primers commonly used for amplification of bacterial 16S rRNA genes. Appl Environ Microbiol. 2008;74: 2461–2470. 10.1128/AEM.02272-07 18296538 PMC2293150

[34] Frank DN, Manigart O, Leroy V, Meda N, Valéa D, Zhang W, et al Altered vaginal microbiota are associated with perinatal mother-to-child transmission of HIV in African women from Burkina Faso. J Acquir Immune Defic Syndr. 2012;60: 299–306. 10.1097/QAI.0b013e31824e4bdb 22343176 PMC6384121

[35] Krebs NF, Sherlock LG, Westcott J, Culbertson D, Hambidge KM, Feazel LM, et al Effects of different complementary feeding regimens on iron status and enteric microbiota in breastfed infants. J Pediatr. 2013;163: 416–423. 10.1016/j.jpeds.2013.01.024 23452586 PMC3674183

[36] Li E, Hamm CM, Gultai AS, Sartor RB, Chen H, Wu X, et al Inflammatory bowel diseases phenotype, C. difficile and NOD2 genotype are associated with shifts in human ileum associated microbial composition. PLoS One. 2012;7: e26284 10.1371/journal.pone.0026284 22719818 PMC3374607

[37] Ramakrishnan VR, Hauser LJ, Feazel LM, Ir D, Robertson CE, Frank DN. Sinus microbiota varies among chronic rhinosinusitis phenotypes and predicts surgical outcome. J Allergy Clin Immunol. 2015 3 25. [Epub ahead of print] 10.1016/j.jaci.2015.02.008 25819063

[38] Wang Q, Garrity GM, Tiedje JM, Cole JR. Naive Bayesian classifier for rapid assignment of rRNA sequences into the new bacterial taxonomy. Appl Environ Microbiol. 2007;73: 5261–5267. 17586664 10.1128/AEM.00062-07 PMC1950982

[39] Robertson CE, Harris JK, Wagner BD, Granger D, Browne K, Tatem B, Feazel LM, Park K, Pace NR, Frank DN. Explicet: graphical user interface software for metadata-driven management, analysis and visualization of microbiome data. Bioinformatics. 2013;29: 3100–3101. 10.1093/bioinformatics/btt526 24021386 PMC3834795

[40] Cohen J. Statistical power analysis for the behavioral sciences. 1st ed New York and London: Academic Press; 1969.

[41] Wagner BD, Robertson CE, Harris JK. Application of two-part statistics for comparison of sequence variant counts. PLoS One. 2011;6: e20296 10.1371/journal.pone.0020296 21629788 PMC3100341

[42] Frank DN, Zhu W, Sartor RB, Li E. Investigating the biological and clinical significance of human dysbioses. Trends Microbiol. 2011;19: 427–434. 10.1016/j.tim.2011.06.005 21775143 PMC3164499

[43] Shukla SK, Murali N, Brilliant M. Personalized medicine going precise: from genomics to microbiomics. Trends Mol Med. 2015 6 27. [Epub ahead of print] 10.1016/j.molmed.2015.06.002 PMC4530069 26129865

[44] Mayer EA, Tillisch K, Gupta A. Gut/brain axis and the microbiota. J Clin Invest. 2015;125: 926–938. 10.1172/JCI76304 25689247 PMC4362231

[45] Maranduba CM, De Castro SB, de Souza GT, Rossato C, de Guia FC, Valente MA, et al Intestinal microbiota as modulators of the immune system and neuroimmune system: impact on host health and homeostasis. J Immunol Res. 2015;2015: 931574 10.1155/2015/931574 25759850 PMC4352473

[46] West CE, Renz H, Jenmalm MC, Kozyrskyi AL, Allen KJ, Vuillermin P, et al The gut microbiota and inflammatory noncommunicable disease: associations and potentials for gut microbiota therapies. J Allergy Clin Immunol. 2015;135: 3–13. 10.1016/j.jaci.2014.11.012 25567038

[47] O’Sullivan O, Cronin O, Clarke SF, Murphy EF, Molloy MG, Shanahan F, et al Exercise and the microbiota. Gut Microbes. 2015;6: 131–136. 10.1080/19490976.2015.1011875 25800089 PMC4615660

[48] Evans CC, LePard KJ, Kwak JW, Stancukas MC, Laskowski S, Dougherty J, et al Exercise prevents weight gain and alters the gut microbiota in a mouse model of high fat diet-induced obesity. PLoS One. 2014;9: e92193 10.1371/journal.pone.0092193 24670791 PMC3966766

[49] Mika A, Van Treuren W, González A, Herrera JJ, Knight R, Fleshner M. Exercise is more effective at altering gut microbial composition and producing stable changes in lean mass in juvenile versus adult male F344 rates. PLoS One. 2015;10: e0125889 10.1371/journal.pone.0125889 26016739 PMC4446322

[50] Lambert JE, Myslicki JP, Bomhof MR, Belke DD, Shearer J, Reimer RA. Exercise training modifies gut microbiota in normal and diabetic mice. Appl Physiol Nutr Metab. 2015;40: 749–752. 10.1139/apnm-2014-0452 25962839

[51] Clarke SF, Murphy EF, O’Sullivan O, Lucey AJ, Humphreys M, Hogan A, et al Exercise and associated dietary extremes impact on gut microbial diversity. Gut. 2014;63: 1913–1920. 10.1136/gutjnl-2013-306541 25021423

[52] Benítez-Páez A, Álvarez M, Belda-Ferre P, Rubido S, Mira A, Tomás I. Detection of transient bacteraemia following dental extractions by 16S rDNA by pyrosequencing: a pilot study. PLoS One. 2013;8: e57782 10.1371/journal.pone.0057782 23469240 PMC3587628

[53] Lee TH, Hsueh PR, Yeh WC, Wang HP, Wang TH, Lin JT. Low frequency of bacteremia after endoscopic mucosal resection. Gastrointest Endosc. 2000;52: 223–225. 10922095 10.1067/mge.2000.107718

[54] Maes M, Coucke F, Leunis JC. Normalization of the increased translocation of endotoxin from gram negative enterobacteria (leaky gut) is accompanied by a remission of chronic fatigue syndrome. Neuro Endocrinol Lett. 2007;28: 739–744. 18063928

[55] Proal AD, Albert PJ, Marshall TG, Blaney GP, Lindseth IA. Immunostimulation in the treatment for chronic fatigue syndrome/myalgic encephalomyelitis. Immunol Res. 2013;56: 398–412. 10.1007/s12026-013-8413-z 23576059

[56] Rao AV, Bested AC, Beauline TM, Katzman MA, Iorio C, Berardi JM, et al A randomized, double-blind, placebo-controlled pilot study of a probiotic in emotional symptoms of chronic fatigue syndrome. Gut Pathog. 2009;1: 6 10.1186/1757-4749-1-6 19338686 PMC2664325

[57] Ianiro G, Bibbò S, Gasbarrini A, Cammarota G. Therapeutic modulation of gut microbiota: current clinical applications and future perspectives. Curr Drug Targets. 2014;15: 762–770. 24909808 10.2174/1389450115666140606111402

